# Comparison of Faster R-CNN, YOLO, and SSD for Third Molar Angle Detection in Dental Panoramic X-rays

**DOI:** 10.3390/s24186053

**Published:** 2024-09-19

**Authors:** Piero Vilcapoma, Diana Parra Meléndez, Alejandra Fernández, Ingrid Nicole Vásconez, Nicolás Corona Hillmann, Gustavo Gatica, Juan Pablo Vásconez

**Affiliations:** 1 Faculty of Engineering, Universidad Andres Bello, Santiago 7500735, Chile; p.vilcapomacarhuamac@uandresbello.edu (P.V.); ggatica@unab.cl (G.G.); 2 Faculty of Dentistry, Universidad de las Américas, Quito 170513, Ecuador; 3 Laboratorio de Odontología Traslacional, Facultad de Odontología, UNAB, Santiago 7591538, Chile; alejandra.fernandez@unab.cl (A.F.); n.coronahillmann@uandresbello.edu (N.C.H.); 4 Centro de Biotecnología Daniel Alkalay Lowitt, Universidad Técnica Federico Santa María, Valparaiso 2390136, Chile; ingrid.vasconez@sansano.usm.cl; 5 Energy Transformation Center, Faculty of Engineering, Universidad Andres Bello, Santiago 7500971, Chile

**Keywords:** dentistry, third molars angle detection, artificial intelligence, convolutional neural networks

## Abstract

The use of artificial intelligence algorithms (AI) has gained importance for dental applications in recent years. Analyzing AI information from different sensor data such as images or panoramic radiographs (panoramic X-rays) can help to improve medical decisions and achieve early diagnosis of different dental pathologies. In particular, the use of deep learning (DL) techniques based on convolutional neural networks (CNNs) has obtained promising results in dental applications based on images, in which approaches based on classification, detection, and segmentation are being studied with growing interest. However, there are still several challenges to be tackled, such as the data quality and quantity, the variability among categories, and the analysis of the possible bias and variance associated with each dataset distribution. This study aims to compare the performance of three deep learning object detection models—Faster R-CNN, YOLO V2, and SSD—using different ResNet architectures (ResNet-18, ResNet-50, and ResNet-101) as feature extractors for detecting and classifying third molar angles in panoramic X-rays according to Winter’s classification criterion. Each object detection architecture was trained, calibrated, validated, and tested with three different feature extraction CNNs which are ResNet-18, ResNet-50, and ResNet-101, which were the networks that best fit our dataset distribution. Based on such detection networks, we detect four different categories of angles in third molars using panoramic X-rays by using Winter’s classification criterion. This criterion characterizes the third molar’s position relative to the second molar’s longitudinal axis. The detected categories for the third molars are distoangular, vertical, mesioangular, and horizontal. For training, we used a total of 644 panoramic X-rays. The results obtained in the testing dataset reached up to 99% mean average accuracy performance, demonstrating the YOLOV2 obtained higher effectiveness in solving the third molar angle detection problem. These results demonstrate that the use of CNNs for object detection in panoramic radiographs represents a promising solution in dental applications.

## 1. Introduction

Nowadays, the advancement of artificial intelligence (AI) has piqued the interest of researchers and programmers in incorporating this technology into numerous fields of dentistry. These advances have encouraged the creation of AI technological tools for activities such as patient management, education, predictive diagnostics, treatment planning, and even the detection of dental micro-organisms [1,2,3,4,5]. The term AI in dentistry applications corresponds to programming algorithms that are developed to fulfill work tasks similar to the performance of a dentist or worker related to specific dentistry activities. In particular, vision algorithms based on AI for dentistry can represent novel solutions to support human decision-making and to improve early diagnosis of pathologies [1,6].

To use AI-based algorithms, it is usually recommended to have a large amount of data that allows the algorithm to be trained and calibrated to fulfill successfully certain dentistry tasks. The dataset (or database) for dentistry applications corresponds to magnetic resonance imaging (MRI), three-dimensional (3D) digital models, red-green-blue (RGB) dental images, or radiographs (X-rays) obtained from diagnoses and medical histories of patients. The combination of an AI-based algorithm with an extensive database has also been demonstrated to be useful during the analysis of CAD/CAM (computer-aided design/computer-aided manufacturing) systems [7,8]. There are multiple initiatives and research found in the literature in which the use of AI in dentistry allowed algorithms to detect a variety of pathologies. The most prevalent are periodontal disease, caries, and tissue lesions. These AI-based algorithms collect and present all this information in results to be later used by specialists as optimization and support tools for dental treatment, personalizing diagnosis, dental prostheses, planning and development of treatments for each patient, and even assistance in oral area surgeries [7,8]. Although this technology has allowed dentists during diagnosis and assistance to solve dental problems, there are still several research challenges in dentistry that keep this research field on a long and open path. For example, the reliability of the algorithm as well as the confidence of the result given by the AI-based algorithm might have high percentages of errors. Therefore, both the treatments and the diagnoses of dentists could damage the oral health and safety of patients because dental specialists need to work in an invasive manner with human patients. Another challenge that is extremely relevant in the implementation of AI-based algorithms is the collection and labeling of data. The database must be sufficiently broad, varied, robust, and correctly classified in labels by dental professionals to develop a concise and reliable AI-based model [7,8]. Finally, errors in training, configuration, or calibration can be responsible for the creation of AI-based models with overfitting (the model only works well with known data) or underfitting problems (the model did not learn well from the data). These problems affect the ability of the AI-based model to interpret new data and achieve generalization capabilities [9,10].

### 1.1. Outline

In Section 1.2, we present the state of the art of the proposed work, as well as a summary of the related works found in the literature in Section 1.3. In Section 2, we present and explain in detail each stage of the proposed methodology for third molar angle detection based on the Faster R-CNN, YOLO V2, and SSD algorithms implementing the architectures of ResNet-18, ResNet-50, and ResNet-101 as feature extraction stages. In Section 4, we present the results of validation and testing using precision-recall curves and other performance metrics. We also made a comparative table of screening results, we assessed bias, variance, overfitting, and underfitting at this stage. Finally, we present the main findings and discussion in Section 4.6, as well as the most important conclusions of our work in Section 5.

### 1.2. State of the Art

**Figure 1:**
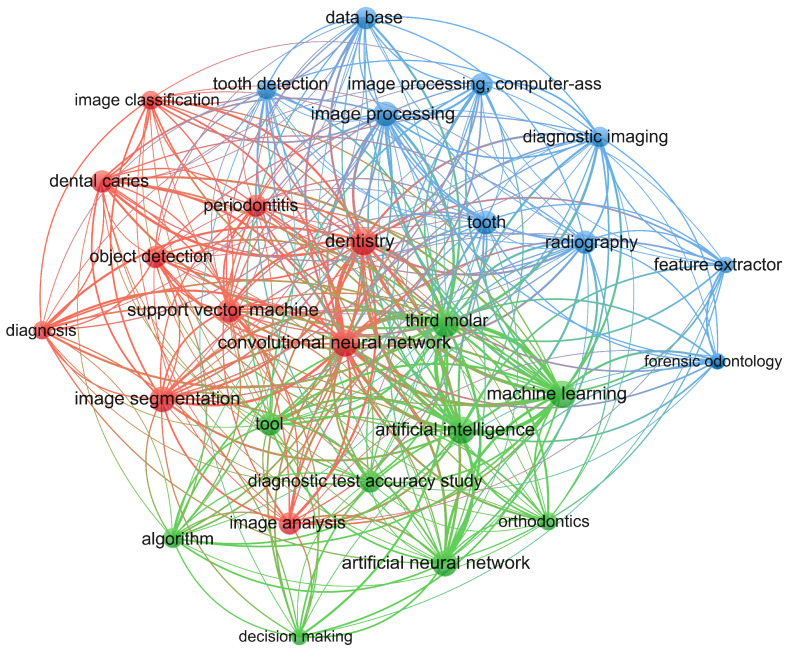
Conceptual map for a search result for third molar detection literature from 2017 to August 2024.

Within the literature, we have found various works and research where AI models were developed aimed at visual detection and classification problems in dentistry. We performed a search in the Scopus database with the keywords “dentistry”, “machine learning”, “deep learning”, and “convolutional neural network” to download a total of 72 documents related to those topics. We used those documents to create the illustration presented in Figure 1 to represent the most commonly used terms and words obtained during the search result.

As can be seen in Figure 1, several topics are considered for research that involves the implementation of AI algorithms in dentistry. The topics that are used most repeatedly are databases, convolutional neuronal networks, machine learning, artificial neural networks, algorithms, feature extractors, image analysis, image segmentation, object detection, dental caries, periodontitis, orthodontics, and diagnosis, among others. It can be seen that several research fields related to AI and dentistry are strongly related to artificial vision systems, highlighting Machine Learning (ML), Deep Learning (DL), and computer vision (CV). We briefly summarize each of those research areas as follows:

- Machine learning (ML) is a subfield of artificial intelligence (AI) that is dedicated to developing algorithms capable of “learning” statistical patterns in data, with the ultimate goal of predicting previously unobserved data [11].
- Deep Learning (DL) is a subset of ML that is based on multi-layer artificial neural networks (ANN). Deep neural networks are especially useful with big datasets, as well as for vision tasks. Often, DL methods excel ML approaches at image segmentation, classification, and detection [2,11,12].
- Computer vision (CV) allows machines to process and interpret data visually. It allows you to address the problems of artificial vision systems since it provides the ability to understand and interpret data. The relevant topics related to this concept are object detection, image classification, and segmentation [12].

**Figure 2:**
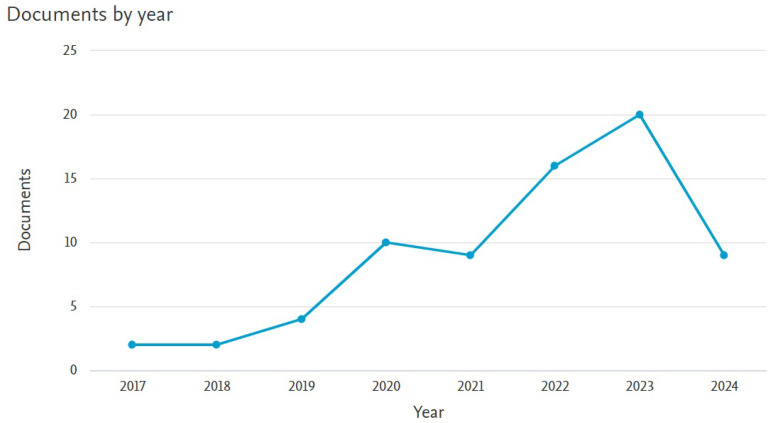
History of published documents based on the implementation of AI in dentistry from 2017 to August 2024.

In addition, we developed an analysis of the state of the art corresponding to the number of documents and their publication history from each year starting from 2017 to 2024, as can be observed in Figure 2. It is to be noticed that the development of AI in dentistry began with two publications in 2017, and that value rose to 20 articles in 2023. In the first half of 2024, we can observe that there are already more documents published than in the first years of research of this analysis.

**Figure 3:**
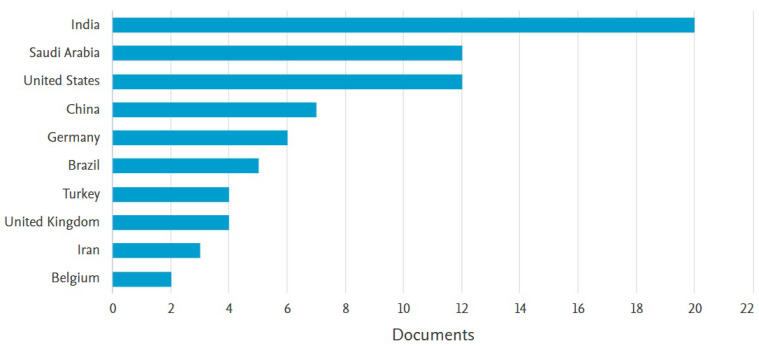
Relationship between published documents related to AI and dentistry and countries of origin from 2017 to August 2024.

Likewise, we carried out an analysis of documents published by each country concerning AI for dentistry, which is presented in Figure 3. As can be seen in the figure, the countries that have the greatest interest in this research topic are India, Saudi Arabia, the United States, China, and Germany. This shows evidence of how AI in the dentistry research field is increasing, especially in developed countries. In the following Section 1.3, we present a summary of the related work that has been developed to date regarding AI and dentistry.

### 1.3. Related Work

In this subsection, we describe in detail the main research works that have been found in the literature related to AI and dentistry. In the work developed by Mahdi, F.P. et al. [13], a Faster R-CNN detection model based on residual networks is used to perform tooth recognition. For this, the ResNet-50 and ResNet-101 neural networks were used to subsequently use a candidate optimization technique based on the positional relationship and the confidence score, achieving results of up to 97.4% for ResNet-50 and values of 98.1% for ResNet-101. When applying the optimization, the results of ResNet-101 range from 97.8% to 98.2%, while in the validation procedure, an accuracy of 97% was achieved. Abdalla-Aslan, R. et al. [14] addressed dental analysis and care as a research topic. For this work, detection and classification algorithms were developed using 83 panoramic X-ray images made up of dental restorations in panoramic radiography. A total of 83 panoramic X-ray images were used to train a model, obtaining results with an accuracy of up to 93.6%. Lee, J.H et al. [15] developed an AI method in conjunction with a computer vision algorithm that combines DL and CNN to carry out dental caries diagnosis and application applications. For this, the GoogleNet and Inception V3 networks were trained and a dataset of 3000 X-ray images was used to evaluate the detector. This method achieved up to 89% accuracy. Similarly, in the paper developed by Zhang, X. et al. [16], an algorithm was developed based on a CNN using a database made up of 3932 RGB images of oral photographs. A computer vision algorithm was developed to detect caries in teeth with sensitivity results of up to 81.90%. In addition, the authors of the study in Moran, M. et al. [17], propose a method that combines image processing and CNNs to achieve the identification of dental caries based on X-rays and classify them according to the severity of the injury. To this end, they used a database made up of 112 radiographs together with data augmentation. The architecture was based on both Inception and ResNet neural networks for the training process, obtaining up to 73.3% accuracy percentages for the test set. Another work used the VGG-16 architecture to develop an automated classification algorithm De Tobel, J. et al. [18]. The authors used a total of 1352 X-ray images to train the model, and the results related to the identification and enumeration of teeth had an accuracy of up to 99.45%. In Yamaguchi, S. et al. [19], a CNN-based algorithm was proposed to obtain the detachment probability of CAD/CAM composite resin cores. The developed algorithm was trained with a set of 8640 3D images, obtaining results with up to 97% accuracy. Another work used a CNN algorithm with XGBoost architecture to train a model from 4135 images of electronic dental records. This approach allowed decisions to be made for tooth extraction with precision percentages of up to 87%. Cyst analysis is another dental application that has used AI as the case in Yang, H. et al. and Lee, J.H et al. [20,21]. In these works, the proposed algorithms were developed by using CNNs such as You Only Look Once (YOLO) architecture. The authors used 1602 panoramic X-ray images to detect keratocysts and odontogenic tumors achieving accuracy results of 66.3%. Other deep algorithms developed for the detection and diagnosis of cystic lesions used deep CNN models. Lee, J.H et al. trained a model with 1140 X-ray images and 986 computed tomography (CTs), obtaining accuracy results of up to 84.7% for X-rays and 91.4% for computerized tomography, respectively [21]. In the work of Banjšak, L. et al. [22], the authors used a CNN for age estimation using a panoramic X-ray database, with 4035 images of living patients and 89 users, achieving results with accuracy of up to 73%. In another work, the authors proposed an algorithm to identify facial reference points for orthodontics based on the YOLO architecture, with accuracy results of 97.7% [23]. However, the authors only trained the algorithms with 22 RGB images, which is probably not enough for a feasible evaluation of this application. In Fu, Q. et al. [24], a DL approach was used to develop an algorithm to detect oral cancer using CNN. For this, a database based on 6176 RGB images is used for training algorithms achieving results of 91% sensitivity, 93.5% efficiency, and 92.3% accuracy. In Schneider, L. et al. [25], different deep learning architectures are references for the segmentation of dental structures in X-rays. A total of 72 models were built by combining six neural network architectures (U-Net, U-Net++, Feature Pyramid Networks, LinkNet, Pyramidal Scene Analysis Network, and Mask Attention Network) with 12 encoders. Three different families (ResNet, VGG, DenseNet) of different depths were used for this work. The design of each model was initiated using three strategies (ImageNet, CheXpert, and random initialization), resulting in 216 trained models. These models were trained for 200 seasons with the Adam optimizer (learning rate = 0.0001) and a batch size of 32, using a dataset of 1625 manually annotated radiologists. A five-fold cross-validation was applied, and performance was mainly quantified by the F1 score. Initialization with ImageNet or CheXpert significantly exceeds random initialization (*p* < 0.05). The authors demonstrated that VGG-based models were more robust in different configurations, while more complex models for the ResNet family achieved higher accuracy results. Finally, in a document selected for the state-of-the-art analysis, a Deep CNN algorithm is proposed and validated to perform the detection and segmentation of the lower third molars and the lower alveolar nerve for dental panoramic X-rays. For this purpose, 81 panoramic X-ray images were used the lower third molars were manually segmented, and the U-net-based DL technique was used to train the convolutional neural network, resulting in an average of 94.7% with an error of 0.3% and 84.7% with an error of 0.9% Vinayahalingam, S. et al. [26].

The works found in the literature demonstrate that several researchers developed different ML, DL, and vision algorithms for dentistry applications and achieved sundry results. The most representative examples are within the research area of diagnosis that comes from detection and classification using DL and vision. Although these investigations exist, the detection of third molar angles remains an open research issue. This is partly due to the difficulty of finding a model that can be adapted to a specific distribution of datasets, which depends on several factors, including the extraction stage of the model characteristics, the configuration of the hyper-parameter, and the specific dental application.

### 1.4. Main Contributions

Considering the state of the art and related work analysis carried out in previous subsections, we briefly present the main contributions of our work as follows.

- We propose the use of a dataset made up of a total of 647 dental panoramic X-rays that were labeled by expert dentists using Winter’s classification criterion. This criterion considers the position of the third molar from the longitudinal axis of the second molar. Based on Winter’s criterion, we were able to characterize the vertical, distoangular, horizontal, and mesioangular angles of the third molars.
- We train, validate, and test DL-based object detection algorithms based on the You Only Look Once V2 (or YOLO V2), Faster Region-Convolutional Neural Network (Faster R-CNN), and Single Shot Multi-box detector (SSD). We compare the performance of each object detection model by using ResNet-18, ResNet-50, and ResNet-101 for the feature extraction stage of each detector.
- We present our results by using precision-recall curves in the proposed dataset. In addition, we evaluated bias, variance, overfitting, and underfitting for each model trained based on YOLO V2, Faster R-CNN, and SSD for the proposed dataset along with detection results and comparison tables of detection percentages.

## 2. Materials and Methods

**Figure 4:**
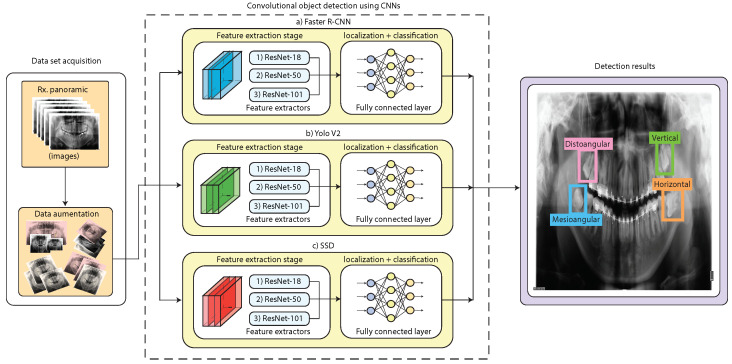
Proposed methodology for third molar angle detection in X-rays using Winter’s criterion. We used Faster R-CNN, YOLO V2, and SSD combined with ResNet-18, ResNet-50, and ResNet-101 feature extractor CNNs.

**Figure 5:**
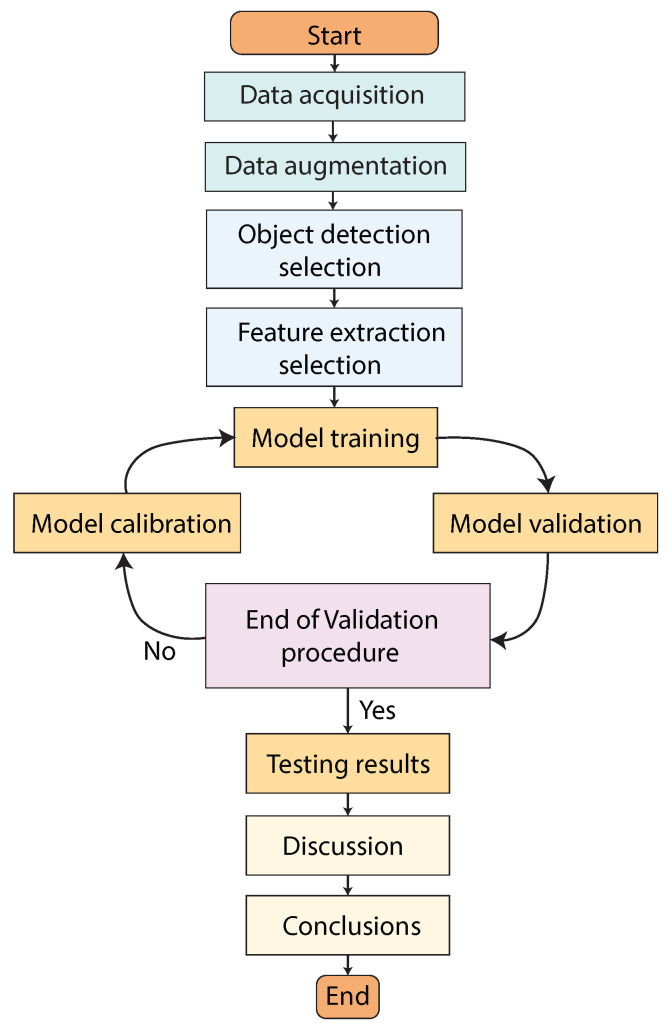
Proposed methodology Workflow for third molar angle detection in X-rays using Winter’s criterion.

In this work, we propose several architectures based on DL object detection algorithms to detect the angle of third molars from dental X-rays using Winter’s criterion. The proposed methodology is represented in Figure 4 and Figure 5. As can be seen, the first stage is the dataset acquisition, in which we explain the X-ray dataset distribution and the data augmentation procedure. In the second stage, we propose the use of Faster R-CNN, YOLO V2, and SSD object detection models. Each object detection model was tested with different CNN-based feature extraction techniques, which are ResNet-18, ResNet-50, and ResNet-101. Finally, in the results stage, we present the training, validation, and testing results and a comparative analysis of the object detection models. The following sections will provide a detailed explanation of each stage of the suggested technique.

### 2.1. Workflow Methodology

The workflow for this study involves several key stages, each critical to the overall process of detecting third molar angles using convolutional neural networks (CNNs) and object detection models. The process can be summarized as follows:

### 2.2. Data Acquisition

**Figure 6:**
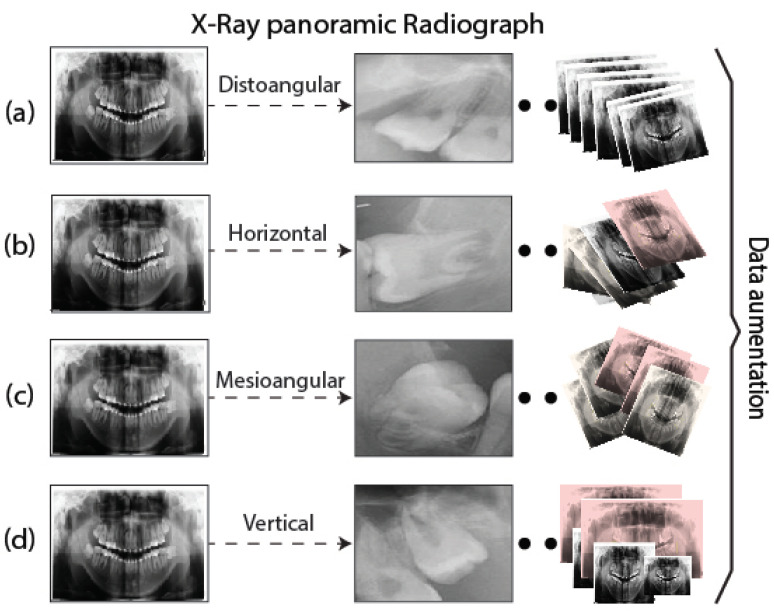
Distribution of third molar angle to Data Acquisition. (**a**) Distoangular, (**b**) Horizontal, (**c**) Mesioangular, (**d**) Vertical.

In this work, we constructed and manually labeled the dataset used for the detection of third molar angles for panoramic dental X-rays based on Winter’s classification criterion [1,27,28]. Such a criterion was used for dentist specialists to label four different categories for the third molar angles, which are as follows: distoangular, horizontal, mesioangular, and vertical [28]. The dataset was then divided into training, validation, and test sets. The proposed database consists of a total of 322 panoramic X-ray images of the third molar at different angles. The images that make up this dataset are standardized to a resolution of 1726×891 pixels. To increase the number of images, three random data augmentation techniques were implemented within the original dataset, such as saturation transformations, changes in brightness, image rotation, and a scalpel in resolution. This last technique consists of changing the resolution of the image to a new format of 600×600 pixels. The rotation involves randomly rotating the image in a range of 0.5 to 10 degrees in the clockwise direction for all original images. Finally, a threshold filter is applied that changes the saturation and brightness of the images. We illustrate the procedure of data augmentation in Figure 6, as well as the detailed distribution of the dataset in Table 1.

The proposed training set is used to train the Faster R-CNN, YOLO V2, and SSD algorithms for third molar angle detection. The validation set is used to calibrate the hyperparameters of the detection models to find the most optimal model that fits the distribution of our dataset. Finally, the efficiency of the detectors was evaluated to determine the best validation found for each model using a new set of panoramic dental X-rays, this allowed us to evaluate the generalization capacity of the proposed models and to check whether the algorithms have any overfitting or underfitting problems.

The research protocol (N 84-2024) received approval from the Ethics-Scientific Committee of the Faculty of Dentistry at Universidad Andrés Bello following Resolution 603/2023 and the updated University decree No. 4522/2022. It is worth mentioning that the data used in our research, and the data of each patient were securely stored and treated with the utmost confidentiality, adhering to strict procedures in data acquisition to maintain integrity and privacy. The panoramic radiographs, collected between January 2022 and December 2024, were anonymized with identifying information removed and replaced with unique codes known only to the research team. Metadata such as age, gender, and clinical notes were securely stored in a database accessible only to authorized personnel, ensuring compliance with data protection regulations. Two specialists in dentistry determined the ground truth for our study, Diana Parra Meléndez from the Faculty of Dentistry, Universidad de las Américas, Quito, Ecuador, and Alejandra Fernández from the Laboratorio de Odontología Traslacional, Facultad de Odontología, UNAB, Santiago, Chile, who meticulously labeled the panoramic radiographs using the MATLAB Image Labeler tool. In total, 322 radiographs and 802 molars were labeled, and this number doubled to 644 radiographs and 1604 molars when using data augmentation. Additionally, Nicolás Corona from the Laboratorio de Odontología Traslacional, Faculty of Dentistry, UNAB, provided valuable support in the collection and labeling of the images.

### 2.3. Third Molar Angle Detection Using CNN-Based Object Detection

In this work, we propose the use of fast and high-performance CNN-based object detection algorithms which are Faster R-CNN, YOLO V2, and SSD. Those algorithms are explained in Section 2.3.1. For each algorithm, we tested different feature extraction CNN methods, which are Resnet-18, Resnet-50, and Resnet-101. The feature extraction methods are explained in Section 2.3.2. These are nine different object detection architectures to train, calibrate, validate, and test.

#### 2.3.1. Object Detection Methods

In this work, we train, calibrate, validate, and test three different object detection techniques based on CNNs, which are Faster R-CNN, YOLOV2, and SSD. We briefly explain each of the object detection methods, as well as each of the feature extraction techniques as follows.

##### Faster-RCNN

Faster R-CNN (Region-based Convolutional Neural Networks) is a CNN-based object detection method that integrates three stages: a feature extraction stage, region proposal networks (RPNs), and a classification stage. The feature extraction stage is in charge of extracting the most important feature maps from the X-ray images. Typically, ResNet or VGG are used for the Faster-RCNN object detection network. However, we used ResNet-18, ResNet-50, and ResNet-101 in this work since it obtained high-performance results for our X-ray dataset distribution. Then, the RPNs, which are based on CNNs, propose regions in which the probability of finding objects of interest is high. The RPNs can simultaneously find and propose multiple region proposals of different geometric sizes and configurations due to the use of anchor boxes. Finally, at the classification stage, the Faster-RCNN uses feed-forward fully connected layers followed by a softmax layer to classify the object category and other feed-forward fully connected to refine the bounding box proposed coordinates (further details in [29,30]).

##### YOLO V2

**Figure 7:**
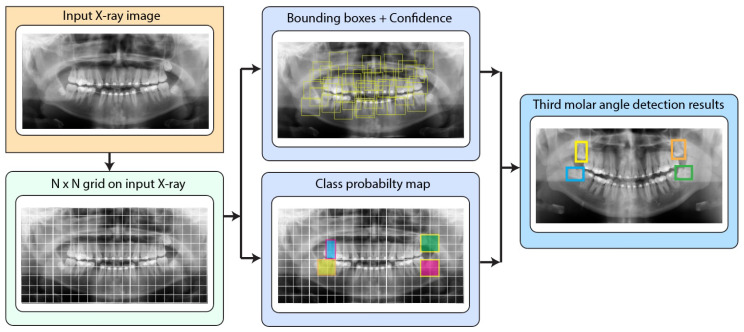
YOLO V2 Pipeline for third molar detection in panoramic X-rays.

YOLO V2 is an object detection method that performs object detection as a single regression problem, directly predicting bounding boxes and class probabilities in a single evaluation. This means that the YOLO V2 does not require an RPN network for work such as in the case of Faster R-CNN. A YOLO v2 algorithm comprises two stages: a feature extraction network and a detection network, as can be obserbed in Figure 7. Typically, YOLO V2 uses a feature extraction stage based on GoogLeNet as an engine for high accuracy and speed. However, in this work, we used ResNet-18, ResNet-50, and ResNet-101 for the YOLO V2 feature extraction stage since those networks obtained high-performance results for our X-ray dataset distribution. The details extracted within the feature extraction stage include both low-level details (borders) and high-level semantics information (shapes and textures) [31,32,33]. YOLO V2 applies a feature extraction method on a grid-based approach that divides the input image into N×N cells, which will be used for detecting objects within its region. However, since different objects can have several geometric configurations, YOLO V2 uses a set of anchor boxes of different sizes, shapes, and scales on each cell to accurately localize all kinds of objects [31,32]. Each anchor box prediction is based on a confidence score that indicates an object’s presence and its category prediction’s accuracy. YOLO V2 uses non-maximum suppression to improve the results and eliminate the repetition of several encompassing boxes for the same object of interest, which improves the accuracy of the detection [31]. Likewise, objects that have lower trust scores are eliminated at a default threshold (for example, 70% confidence), which improves the final result. The output of YOLO v2 includes encompassing boxes that are labeled with their respective categories of objects and scores that indicate the probability that the object detected corresponds to its category.

##### SSD

The last object detection method we used in our work is the Single Shot MultiBox Detector (SSD). This object detection technique is usually known for its low inference time, which is reached by eliminating the need for a region proposal stage used in Faster R-CNN. The SSD uses a single-shot approach where both the object localization and classification tasks are performed simultaneously. Typically, the SSD algorithm uses a feature extraction stage composed of CNNs such as VGG16. Nevertheless, we employed ResNet-18, ResNet-50, and ResNet-101 in our study due to their exceptional performance on our panoramic X-ray dataset distribution. The next stage in the SDD algorithm combines additional convolutional layers to enable the detection of objects at multiple scales by producing predictions from feature maps of varying resolutions. SDD also uses a set of anchor boxes with varying aspect ratios and scales at each feature map cell, allowing the network to detect objects of various sizes and geometrical configurations. For each anchor box, SSD predicts both a category score and the offsets for the bounding box to better localize the objects of interest. By applying convolutional operations across the anchor boxes on different feature layers, SSD can simultaneously handle object localization and classification problems [34,35].

#### 2.3.2. Feature Extraction Methods

In this work, we propose the use of three CNN-based models as feature extractors that are able to work fast and have high performance on our X-ray dataset distribution. The selected feature extraction networks are ResNet-18, ResNet-50, and ResNet-101. Compared to the rest of the CNNs, the main architecture of ResNet layers is based on residual layers that allow the creation of connections that add the input directly to the output [36]. This structure facilitates the flow of gradients through the network during the backpropagation process to facilitate network training and helped us obtain outstanding results for third-molar angle detection. We explain in detail the feature extraction methods used in this work as follows.

##### ResNet-18

ResNet-18 is a CNN with 11.7 million parameters that address the vanishing gradient problem by using residual layers [36,37]. The convolutional layers, batch normalization, and Relu activation functions compose the entire 18-layer architecture of ResNet-18. This network has an input resolution of 224×224×3; therefore, any image different from that resolution should be resized. The rest of the layers that compose ResNet-18 correspond to Relu layers, batch normalization layers, residual layers, fully connected layers, and the softmax for classification. In our case, the categories of interest for classification correspond to the third molar angle (vertical, distoangular, horizontal, and mesioangular). The structure of the ResNet-18 network begins with a 7×7 convolutional layer with 64 filters (stride of 2), followed by batch normalization, a ReLU activation function, and a 3×3 max-pooling layer (stride of 2) [38,39]. The max-pooling layer helps to reduce the size of the spatial dimensions of the input panoramic X-ray images, which allows ResNet-18 to extract patterns from panoramic X-ray images. The final characteristics are sent through a fully connected layer and a softmax layer to obtain the classification results. Further details for ResNet-18 can be found in [38,39,40]. Although ResNet-18 has a structure capable of achieving high-performance accuracy for classification problems, it needs to be combined with the Faster R-CNN, YOLO, and SSD detection models to solve the proposed object detection problem.

##### ResNet-50

ResNet-50 is a CNN that extends the depth and capacity of ResNet-18 by using bottleneck residual blocks [41]. ResNet-50 has 25.6 million parameters for training, with an input size of 224×224×3. ResNet-50 starts with a 7×7 convolutional layer with 64 filters (with a stride of 2). Then, batch normalization followed by ReLU and a 3×3 max-pooling are used. ResNet-50 is primarily composed of four stages, each containing bottleneck residual blocks resulting in a cumulative of 50 layers. Each bottleneck block includes a 1×1 layer that reduces the dimensionality (bottleneck), a 3×3 layer that performs the convolution, and another 1×1 layer that restores the dimensionality. These blocks also use batch normalization and ReLU activations after each convolution. ResNet-50 uses shortcut connections that skip one or more layers, allowing gradients to flow directly through the network, thus mitigating the vanishing gradient problem and enabling the training of very deep networks. After the residual stages, a global average pooling layer reduces the spatial dimensions to 1×1, followed by a fully connected layer with four neurons and a softmax activation for third molar angle classification in X-ray images [41,42]. Further details of ResNet-50 can be found in [41,42,43]. Although ResNet-50 has a structure capable of achieving high-performance accuracies for classification problems, it must be combined with the Faster R-CNN, YOLO, and SSD detection models to solve object detection problems.

##### ResNet-101

Resnet-101 is a CNN that uses residual learning such as its ResNet-18 and ResNet-50 predecessors, which allows the training of deep networks while addressing the problem of gradient vanishing. Resnet-101 has 101 layers, which means a greater depth than Resnet-18 (18 layers) and Resnet-50 (50 layers). This allows Resnet-101 to extract more rich features from X-ray images to detect third molar angles using Winter’s criterion. However, this strongly depends on the dataset size and distribution. Resnet-101, as well as Resnet-50, uses bottleneck blocks that consist of convolutional layers of 1×1, 3×3, and 1×1. These bottleneck blocks are more efficient in the parameters compared to the basic residual blocks used in Resnet-18, which consist of two 3×3 convolutions. However, Resnet-101 has a greater number of blocks of this nature, which further expands its capacity. The structure of this neural network is made of 44.6 million parameters available for training [43]. Unlike previous feature extractors, Resnet-101 architecture has 101 deep layers which maintain the structure of the combination and connection of 105 convolutional layers, 105 batch normalization layers, and 100 Relu activation layers. Resnet-101 has an input layer of 224×224×3 but its structure continues with the same number of layers that make up the residual layers similar to the structure of ResNet-50 (further details in [44]). The final features obtained by Resnet-101 are sent through a fully connected layer with four neurons and a softmax layer to obtain the ranking results [45]. Further details of ResNet-50 can be found in [43,44,45]. Although ResNet-101 has a structure capable of achieving very high-performance accuracies for classification problems, it must be combined with the Faster R-CNN, YOLO, and SSD detection models to solve object detection problems.

## 3. Evaluation Metrics

To evaluate the performance of the Faster R-CNN, YOLO, and SSD object CNNs for third molar angle detection in dental panoramic X-rays are necessary to use evaluation metrics such as the Precision-Recall (PR) Curve and the Mean Average Precision (mAP). We explain in detail the PR curves calculation as follows.

### 3.1. Precision and Recall

**Figure 8:**
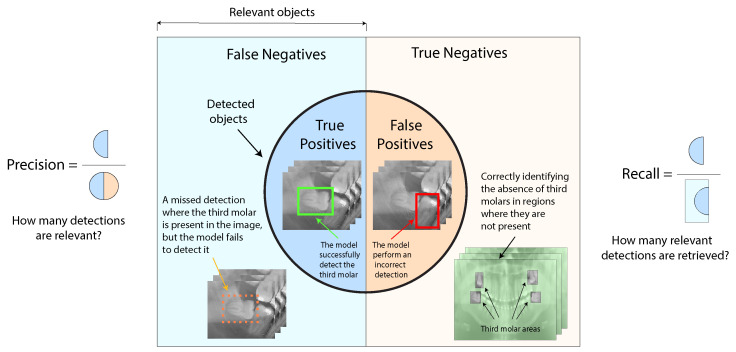
Precision and recall representation for the third molar angle detection in dental panoramic X-rays context.

Precision, also known as positive predictive value (PPV), refers to the proportion of relevant instances among the detected objects within the panoramic X-rays. Recall, usually referred to as sensitivity, is the proportion of relevant instances that were successfully detected within the panoramic X-rays. We define how to calculate precision in Equation (1) and recall in Equation (2), respectively. In addition, we illustrate the precision and recall metrics for the third molar angle detection in the dental panoramic X-rays context in Figure 8.

(1) $$\mathrm{Precision}=\frac{TP}{TP+FP}$$

(2) $$\mathrm{Recall}=\frac{TP}{TP+FN}$$

where TP denotes true positives, FP false positives, and FN false negatives.

### 3.2. Precision-Recall Curve

**Figure 9:**
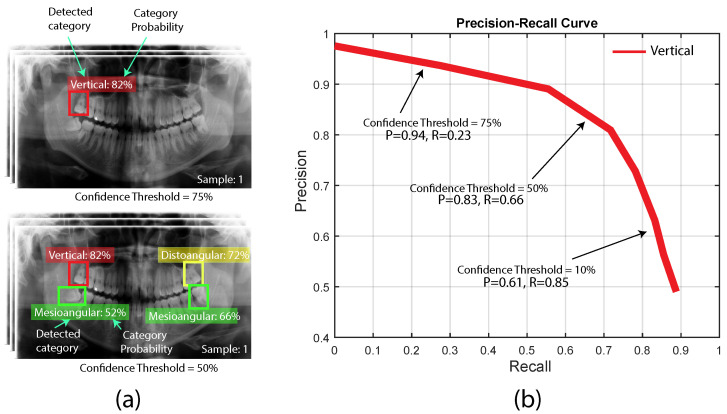
Precision-Recall (PR) Curve for the third molar angle detection in dental panoramic X-rays context. (**a**) Results for the third molar angle detection at different threshold values. (**b**) A sample of the Precision-Recall (PR) Curve for one category (vertical).

The Precision-Recall (PR) Curve shows the trade-off between precision and recall for different threshold configurations for each detected category. It is widely used for object detection problems, which are considered to have imbalanced datasets since the number of true positives (e.g., third molars in X-ray images) is much smaller than the number of true negatives (all the areas where the third molars are not present in X-ray images). To obtain the PR Curve, we first need to vary a decision threshold for the object detection method. Then, we compute the precision and recall for each threshold and plot precision on the *y*-axis and recall on the *x*-axis. A sample of a PR curve for the third molar angle detection in dental panoramic X-ray context can be observed in Figure 9. It should be noted that the confidence value (probability) of each detection is influenced by the confidence threshold used to determine whether it is considered a positive prediction.

### 3.3. Mean Average Precision (mAP)

Mean Average Precision (mAP) is a metric employed to assess the precision-recall performance of object detection models. It offers a singular measure that encompasses the balance between precision and recall at different threshold levels. To calculate the mAP, we first calculate the Average Precision (AP) for each category, which involves integrating the area under the PR Curve. Then, we compute the mean of the Average Precision values across all categories (vertical, distoangular, horizontal, and mesioangular angles of the third molars). The equation for Average Precision (AP) is presented in Equation (3), and the Mean Average Precision (mAP) in Equation (4), respectively.

(3) $$\mathrm{AP}=\sum_{n}\left(R_{n}-R_{n-1}\right)P_{n}$$

where $P_{n}$ and $R_{n}$ are the precision and recall at the *n*-th threshold, respectively.

(4) $$\mathrm{mAP}=\frac{1}{N}\sum_{i=1}^{N}{\mathrm{AP}}_{i}$$

where *N* is the number of classes (in this case, one class for third molar detection).

### 3.4. Inference Times

Inference times refer to the duration of an artificial intelligence model (AI) to process the input data and produce an output prediction. In the context of dental applications that use CNNs for the detection of third molars, inference times are crucial to evaluate the practicality and efficiency of the model in real-world scenarios. Faster inference times mean that dental professionals can quickly obtain diagnostic information from panoramic radiographs, facilitating rapid medical decisions and the early diagnosis of dental pathologies. In our study, we compare the inference times of three Faster R-CNN, YOLO V2, and SSD, together with different feature extraction CNN: ResNet-18, ResNet-50, and ResNet-101.

## 4. Results

This section shows the results of the YOLO V2, Faster R-CNN, and SSD object detection models to localize and characterize the vertical, distoangular, horizontal, and mesioangular angles of the third molars in panoramic X-rays based on Winter’s criterion. Each object detection model was trained and evaluated using three different feature extraction CNNs, which are ResNet-18, ResNet-50, and ResNet-101. We performed training, validation, and testing procedures for each model. We also analyze bias, variance, overfitting, and underfitting. The training, validation, and testing procedures are explained in detail below.

First, we performed a validation procedure by testing several hyper-parameter configurations to train each of the proposed algorithms for the third molar angle detection on panoramic X-rays. We set different values of epochs, learning rate, and different optimizer methods as can be observed in Table 2. We used values of 20 and 40 epochs as the limit of training epochs along with learning rate values of 0.001, 0.005, and 0.0001. We used three optimizers: Adam, SGDM, and RMSProp. Adam adapts learning rates using both momentum and squared gradients, SGDM uses momentum to accelerate convergence, and RMSProp adjusts learning rates based on the mean square of the gradients.

Once the different proposed models were trained using the hyper-parameter configuration of Table 2, we evaluate bias, variance, overfitting, and underfitting considering the following guidelines. If the error in training data is high, then the model has a high bias. If the error in the validation/testing data is much greater than the error in the training data, the model has high variance. If the error in the training and validation data is low and the error in the testing data is significantly higher, the model has overfitting. Finally, if the error in the training, validation, and testing data is high, the model is considered underfitting, which means that the model complexity is not enough to fit the dataset distribution. Considering these guidelines, we analyze our results below in detail.

### 4.1. Results for Third Molar Angle Detection with ResNet-18

In this section, we present the training, validation, and testing results for algorithms using ResNet-18 as a feature extractor of each object detection method as can be observed in Table 3.

**Figure 10:**
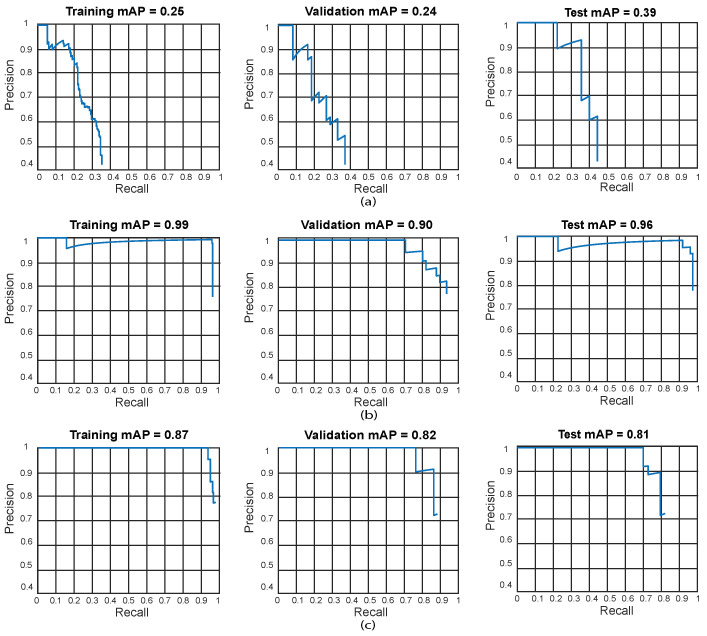
Best precision-recall curves for the obtained algorithms using ResNet-18. Training results (**left**), validation results (**center**), and testing results (**right**). (**a**) Test 2—Faster R-CNN, (**b**) Test 10—YOLO V2, (**c**) Test 12—SSD. YOLO V2 using ResNet-18 obtained the best result in test 10 (training: 99%, validation: 90%, and testing: 96%).

We also present the training, validation, and testing best results by using precision-recall curves as can be observed in Figure 10.

**Figure 11:**
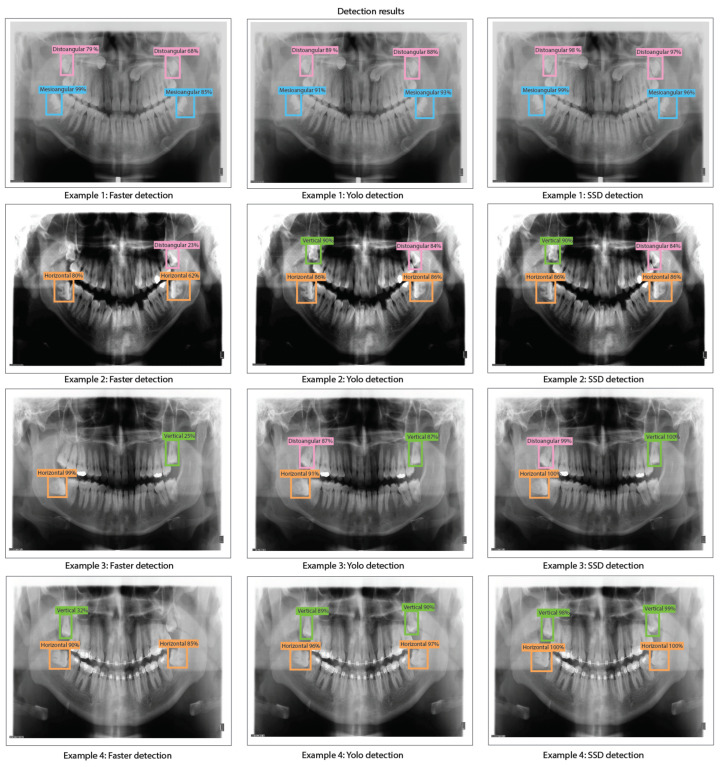
Third molar angle detection in dental panoramic X-rays with ResNet-18 as feature extractor for Faster R-CNN, YOLO v2, and SSD.

The object detection results for Faster R-CNN, YOLO v2, and SSD with ResNet-18 obtained when evaluating the dental panoramic X-rays with the trained algorithms can be observed in Figure 11.

We summarize the main findings of the object detection results for ResNet-18 as feature extraction as follows.

- It can be observed in Table 3 that a few Faster R-CNN, YOLO v2, and SSD models based on ResNet-18 as a feature extractor reach a very low accuracy result. For Faster R-CNN, the test with the lowest results is Test 3 (training: 1.8%, validation: 1.2%, and testing: 1.1%). YOLO V2 also presents low accuracy results in Test 6 (training: 7%, validation: 12%, and testing: 5%). Finally, SSD also presents low results in Test 15 (training: 2.9%, validation: 2%, and testing: 2%). This suggests that such models have an underfitting problem, which means that the models cannot fit the proposed dental panoramic X-rays dataset distribution. Moreover, high error in the training set indicates that the models do not fit well with the dental panoramic X-rays dataset distribution, which means a high bias.
- For Test 16, the Faster R-CNN achieves an accuracy of 18% on the training set, whilst the YOLO V2 and SSD reach 99% and 89%, respectively. This might be because the Faster R-CNN is characterized by using an RPN (Region Proposal Network) stage, which for our application, cannot fit the X-ray dataset distribution (underfitting). On the other hand, YOLO V2 and SSD use a single-stage detection approach without using an RPN stage, which fits better for the proposed X-ray dataset. The results for YOLO V2 and SSD are also high for validation, with 85% and 80%, respectively, and testing, with 95% and 79%, respectively. The high training accuracy of YOLO V2 (99%) and SSD (89%) suggests that these models have a low bias compared to Faster R-CNN. However, the slight drop in validation accuracy (YOLO V2: 85%, and SSD: 80%) and tests for both models (YOLO V2: 95%, and SSD: 79%) indicates a light variance, since the performance of the models decreases slightly in validation and testing experiments.
- We obtained the best results for Faster R-CNN using ResNet-18 were reached in test 2 (training: 25%, validation: 24%, and testing: 39%). For YOLO V2 using ResNet-18, the best result is test 10 (training: 99%, validation: 90%, and testing: 96%). Finally, for the SSD model using ResNet-18, the best result was in test 12 (training: 87%, validation: 82%, and testing: 81%). This indicates that the best model for this experiment set is YOLO V2 using ResNet-18 with the hyper-parameter configuration of test 10. This model has a low bias and variance, demonstrating high generalizing capabilities (no overfitting) since the accuracy is barely reduced from training to validation and testing. We can observe the training, validation, and testing results for models obtained in tests 2, 10, and 12 in Figure 10, in which we present the precision-recall curves for such models.

### 4.2. Results for Third Molar Angle Detection with ResNet-50

In this section, we present the training, validation, and testing results for algorithms using ResNet-50 as a feature extractor as can be observed in Table 4.

**Figure 12:**
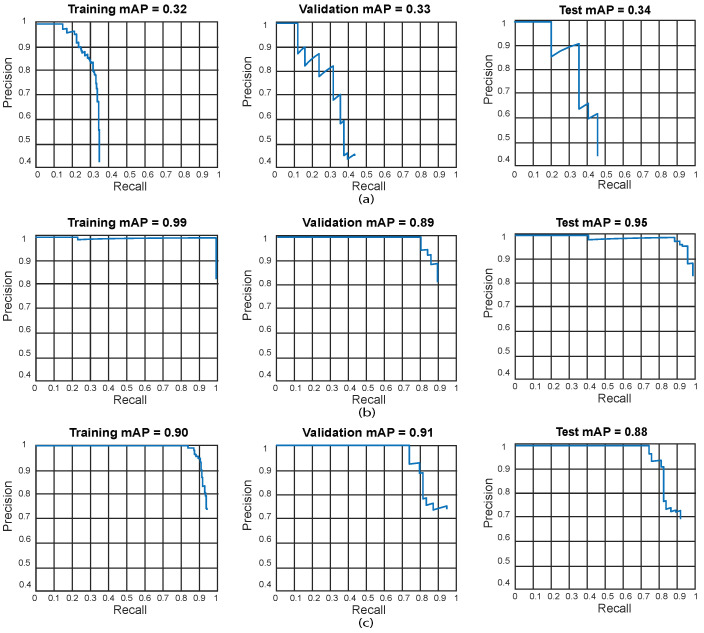
Best precision-recall curves for the obtained algorithms using ResNet-50. Training results (**left**), validation results (**center**), and testing results (**right**). (**a**) Test 11—Faster R-CNN, (**b**) Test 18—YOLO V2, (**c**) Test 9—SSD.

We also present the training, validation, and testing best results by using precision-recall curves as can be observed in Figure 12.

**Figure 13:**
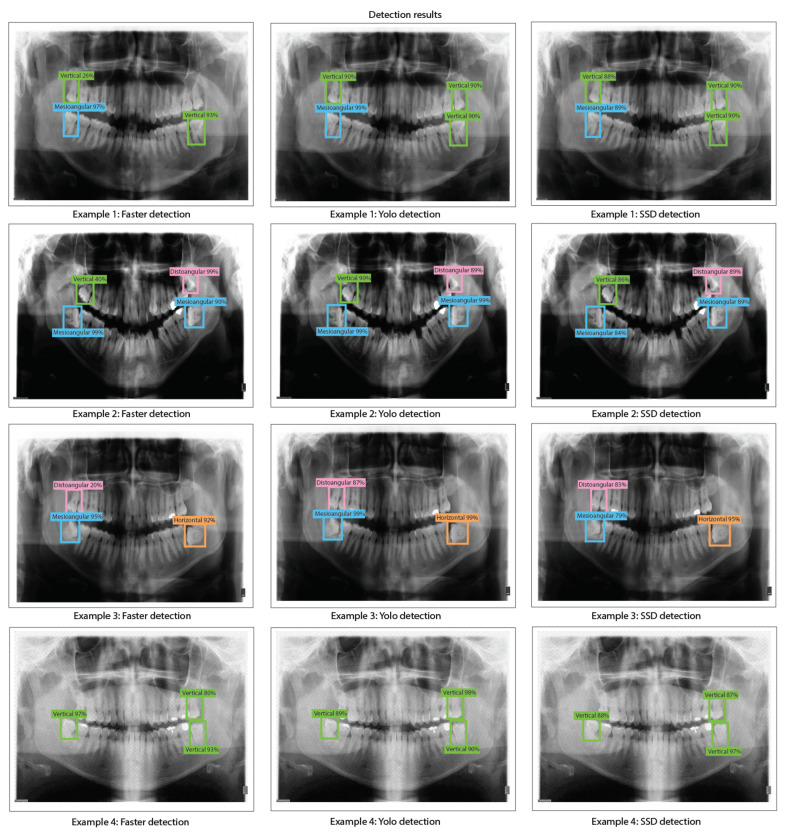
Third molar angle detection in dental panoramic X-rays with ResNet-50 as feature extractor for Faster R-CNN, YOLO V2, and SSD.

The object detection results for Faster R-CNN, YOLO v2, and SSD with ResNet-50 as feature extraction obtained when evaluating the dental panoramic X-rays with the trained algorithms can be observed in Figure 13.

We summarize the main findings of the object detection results for ResNet-50 as feature extraction as follows.

- It can be observed in Table 4 that a few Faster R-CNN, YOLO v2, and SSD models based on ResNet-50 as the feature extractor reach a very low accuracy result. For Faster R-CNN, the test with the lowest results is Test 6 (training: 1.2%, validation: 1.2%, and testing: 1.1%). YOLO V2 also presents low accuracy results in Test 6 (training: 1.8%, validation: 1.5%, and testing: 1.5%). Finally, SSD also presents low results in Test 6 (training: 1.5%, validation: 1.3%, and testing: 1.4%). This suggests that such models have an underfitting problem, which means that the models cannot fit the proposed dental panoramic X-rays dataset distribution. Moreover, a high error in the training set indicates that the models do not fit well with the dental panoramic X-rays dataset distribution, which means a high bias.
- For all the tests, the Faster R-CNN achieves very low accuracy results. The worst results for Faster R-CNN are for test 6 (training: 1.2%, validation: 1.2%, and testing: 1.1%), whilst the best result is for test 11 (training: 35%, validation: 33%, and testing: 34%), which is still a very low accuracy result. This means Faster R-CNN working with ResNet-50 as feature extraction tends to suffer from underfitting for our X-ray dataset distribution. Again, this might be because the Faster R-CNN is characterized by using an RPN (Region Proposal Network) stage.
- We obtained the best results for YOLO V2 using ResNet-50 in test 18 (training: 99%, validation: 89%, and testing: 95%). For the SSD model using ResNet-50, the best result was in test 9 (training: 88%, validation: 91%, and testing: 88%). This indicates that the best model for this set of experiments is YOLO V2 using ResNet-50 with the hyper-parameter configuration of test 18. This model has a low bias and variance, demonstrating high generalizing capabilities (no overfitting) since the accuracy is barely reduced from training to validation and testing. We can observe the training, validation, and testing results for models obtained in tests 11, 18, and 9 in Figure 12 in which we present the precision-recall curves for both models.

### 4.3. Results for Third Molar Angle Detection with ResNet-101

In this section, we present the training, validation, and testing results for algorithms using ResNet-101 as a feature extractor as can be observed in Table 5.

**Figure 14:**
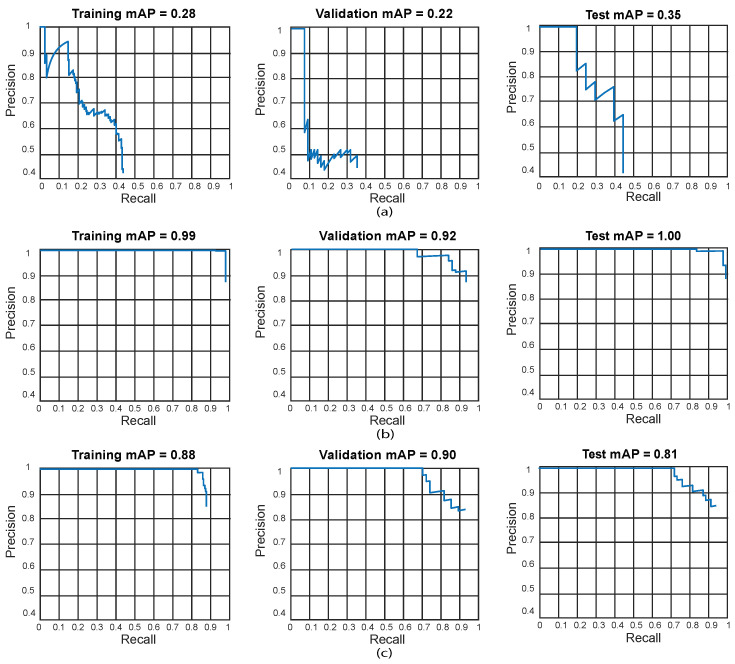
Best precision-recall curves for the obtained algorithms using ResNet-101. Training results (**left**), validation results (**center**), and testing results (**right**). (**a**) Test 5—Faster R-CNN, (**b**) Test 11—YOLO V2, (**c**) Test 12—SSD.

We also present the training, validation, and testing best results by using precision-recall curves as can be observed in Figure 14.

**Figure 15:**
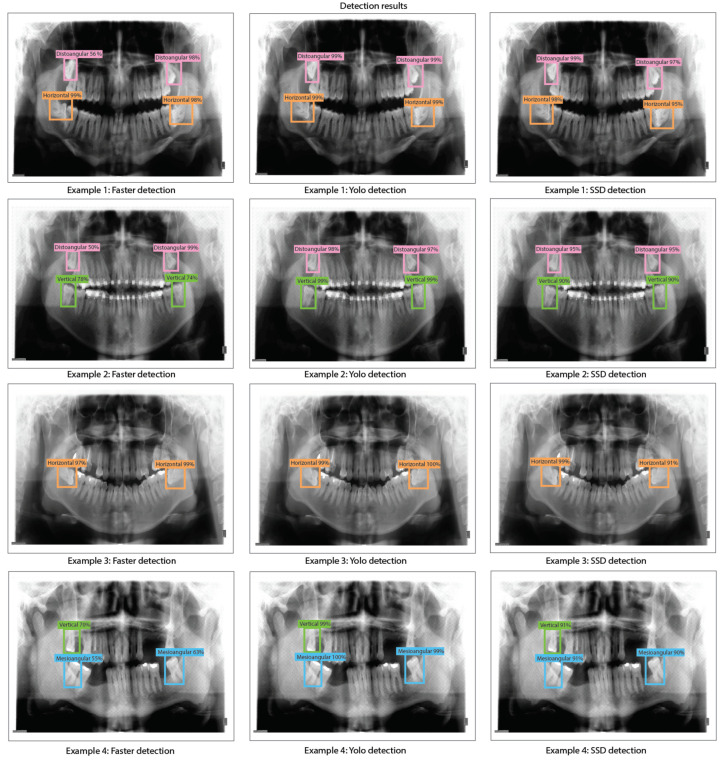
Third molar angle detection in dental panoramic X-rays with ResNet-101 as feature extractor for Faster R-CNN, YOLO V2, and SSD.

The object detection results for Faster R-CNN, YOLO v2, and SSD with ResNet-101 obtained when evaluating the dental panoramic X-rays with the trained algorithms can be observed in Figure 15.

We summarize the main findings of the object detection results for ResNet-101 as feature extraction as follows.

- It can be observed in Table 5 that a few Faster R-CNN, YOLO v2, and SSD models based on ResNet-101 as a feature extractor reach a very low accuracy result. For Faster R-CNN, the test with the lowest results is Test 6 (training: 1.6%, validation: 1.3%, and testing: 1.1%). YOLO V2 also presents low accuracy results in Test 6 (training: 7%, validation: 12%, and testing: 5%). Finally, SSD also presents low results in Test 6 (training: 3.3%, validation: 7.7%, and testing: 2%). This suggests that such models have an underfitting problem, which means that the models cannot fit the proposed dental panoramic X-rays dataset distribution. Moreover, a high error in the training set indicates that the models do not fit well with the dental panoramic X-rays dataset distribution, which means a high bias.
- For all the tests, the Faster R-CNN achieves very low accuracy results. The worst results for Faster R-CNN are for test 6 (training: 1.6%, validation: 1.3%, and testing: 1.1%), whilst the best result is for test 5 (training: 28%, validation: 22%, and testing: 35%), which is still a very low accuracy result. This means Faster R-CNN working with ResNet-101 as feature extraction tends to suffer from underfitting for our X-ray dataset distribution. Once again, this might be because the Faster R-CNN is characterized by using an RPN (Region Proposal Network) stage.
- We obtained the best results for YOLO V2 using ResNet-101 in test 11 (training: 99%, validation: 92%, and testing: 99%). For the SSD model using ResNet-101, the best result was in test 12 (training: 88%, validation: 90%, and testing: 89%). This indicates that the best model for this set of experiments is YOLO V2 using ResNet-101 with the hyper-parameter configuration of test 11. This model has a low bias and variance, demonstrating high generalizing capabilities (no overfitting) since the accuracy is barely reduced from training to validation and testing. We can observe the training, validation, and testing results for models obtained in tests 5, 11, and 12 in Figure 14 in which we present the precision-recall curves for both models.

### 4.4. Comparison of Inference Times

In this subsection, we briefly summarize in Table 6 the inference times for each object detection algorithm (Faster R-CNN, YOLO V2, and SSD) with each of the feature extraction methods (ResNet-18, ResNet-50, and ResNet-101). It is to be noticed that the Faster R-CNN has higher inference times compared to YOLO V2 and SSD. On the other hand, YOLO and SSD have similar inference times. This is because the Faster R-CNN uses an RPN (Region Proposal Network) stage, which requires more time to make an inference. On the other hand, YOLO V2 and SSD use a single-stage detection approach without using an RPN stage, which makes them a faster solution compared to Faster R-CNN [29,46].

### 4.5. Comparison Results with State of the Art Works

In this subsection, we present a comparative analysis in Table 7, in which our work contribution is compared with other state-of-the-art works that have been developed in similar scenarios related to dental applications in third molar angle detection or similar using X-rays (Rx). It is to be noticed that we are the only work in the literature that uses three different object detection methods (Faster, YOLO, and SSD), as well as three different feature extraction methods (ResNet-18, ResNet-50, ResNet-101).

### 4.6. Discussion

In this section, we present a summary of the most important findings and future work insights related to the development of this work.

- During the implementation of third-molar detection algorithms using different detection models and ResNet architectures, it can be observed that YOLO V2 is the model that stands out in terms of both accuracy and consistency during the training, validation, and testing stages. Therefore, the best results from our experiments were obtained by YOLO V2 using ResNet-18 (training: 99%, validation: 90%, and testing: 96%), YOLO V2 using ResNet-101 (training: 99%, validation: 92%, and testing: 99%), and YOLO V2 using ResNet-101 (training: 99%, validation: 92%, and testing: 99%), which indeed are very similar. These high accuracy results indicate that YOLO V2 is very effective for detecting third molar angles in our X-ray dataset distribution. On the other hand, the Faster R-CNN models with ResNet CNNs (ResNet-18, ResNet-50, and ResNet-101) possess underfitting problems for our X-ray dataset distribution. This might be because the Faster R-CNN is characterized by using an RPN (Region Proposal Network) stage. On the other hand, the SSD detection model has moderate accuracy across all ResNet architectures. Although SSD does not achieve the accuracy levels of YOLO V2, it produces reasonably consistent results, making it suitable for applications that require a balance of accuracy and detection speed (inference times less than 100 ms).
- Although each of the ResNet architectures used has several parameters, it is to be noticed that there is no direct correlation between the number of parameters in the ResNet architectures and the detection accuracy obtained. We did not find a direct correlation between the number of parameters and the precision obtained. Although ResNet-50 and ResNet-101 have more parameters and offer superior modeling capability, YOLO V2 with ResNet-18, which has the fewest parameters, still achieves very high accuracy. This might be due to our dataset structure and size. This behavior might change if the dataset increases to the order of thousands or even millions of images since it is well known that while deeper, the network learns better from big datasets. In future works, we will try to increase our dataset distribution for this problem.
- Overall, we have demonstrated in this work that CNN-based object detection techniques can be used to detect third-molar angle detection based on Winter’s classification criterion. The categories that we detected are distoangular, vertical, mesioangular, and horizontal. To detect these categories, we have implemented several object detection methods, which are YOLO V2, Faster R-CNN, and SSD. Each of the mentioned object detection methods was trained by using ResNet-18, ResNet-50, and ResNet-101 as feature extraction methods, in which YOLO V2 with ResNet-18 obtained the best results (training: 99%, validation: 90%, and testing: 96%). These findings represent a promising solution for dentistry applications, such as improved diagnostic accuracy, more automated dental procedures, and even a possible training tool for educational applications. Moreover, we have presented a significant improvement compared to other works, that only train a single object detection method for different applications such as third molar detection and angle detection, tooth identification, caries detection, dental implant detection, and crown detection (see Table 7).
- Despite the promising results obtained, our study has several limitations. First, the dataset used only 644 panoramic X-rays, which may not be large enough to generalize the findings to a broader population. The performance of the models, particularly those with deeper architectures such as Resnet-50 and Resnet-101, can improve with larger datasets. Second, the structure and specific characteristics of the dataset could have influenced the observed results, which limits the applicability of our findings to other types of radiographic images or different dental pathologies. Another problem that will be addressed in the future is the analysis of data from other countries than Chile since other countries’ demographic data might be slightly different for this application. Finally, both YOLO V2 and SSD demonstrated high precision for molar detection. However, its computational complexity and the fact that it is worked based on CNNs makes the use of graphic cards that have parallel processing capacity important for this type of application. This can be an important computational limitation if you want to implement these technologies in real applications in clinics with X-ray equipment already installed.
- Based on the times of inference presented, YOLO V2 demonstrates a strong potential for real-time clinical applications, with inference times that vary from 0.071 to 0.092 s in different Resnet architectures. This positions it well inside the desirable threshold of fewer than 0.3 s (real-time) for quick decision-making in clinical environments. SSD is also promising, with inference times between 0.077 and 0.104 s, so it is a viable option for scenarios that require rapid responses. In contrast, Faster R-CNN is not faster, with inference times from 0.188 to 0.360 s, it is less suitable for real-time use due to its slower processing speed, and in our case, it also presents a poor performance in detection. In general, Yolo V2 stands out as the most effective model for the detection of third molar angles in real-time in clinical environments.
- In this work, we use Resnet-18, Resnet-50, and Resnet-101 for the proposed detection models due to their proven performance and compatibility with Faster R-CNN, Yolo, and SSD. Although we performed preliminary tests with other architectures such as Googlenet, Alexnet, and Inception, Resnet consistently showed outstanding results. In future works, we will perform extensive tests and hyperparameter calibration for extractors of additional characteristics to further improve the precision and reliability of our diagnostic tools based on AI.

## 5. Conclusions

In this work, we proposed an artificial intelligence algorithm based on the Faster R-CNN, YOLO V2, and SSD as object detection models for Third Molar Angle Detection in Dental Panoramic X-rays. For each object detector, we tested different CNN architectures (ResNet-18, ResNet-50, and ResNet-101) as a feature extractor to carry out the third molar angle detection. We used Winter’s criterion to characterize distoangular, vertical, mesioangular, and horizontal third molar angles in panoramic X-ray images. A data augmentation process was carried out to expand the data to build a dataset of greater robustness and increase data variability. We perform training, validation, and testing results for different hyperparameter configurations. We also analyzed bias, variance, overfitting, and underfitting based on the training, validation, and testing results for each model. The best results from our experiments were obtained by YOLO V2 using ResNet-18 (training: 99%, validation: 90%, and testing: 96%), YOLO V2 using ResNet-101 (training: 99%, validation: 92%, and testing: 99%), and YOLO V2 using ResNet-101 (training: 99%, validation: 92%, and testing: 99%), which indeed are very similar. These results offer promising solutions for dental applications, which can range from enhanced diagnostic precision to increased automation of dental procedures. Additionally, we have demonstrated a notable improvement over previous studies, which typically focus on training a single object detection method, or are focused on other tasks such as detecting third molars and their angles, tooth and caries detection, and dental implants and crowns detection. In future works, we will extend the dataset to improve the proposed method’s generalization capabilities. Moreover, additional algorithms such as Mask-RCNN or Vision Transformers (ViTs) can be implemented in the future to analyze their results in our dataset distribution.

## Figures and Tables

**Table 1:** Dataset distribution for X-ray panoramic images.

	Data		
Database	Training	Validation	Testing
Normal	258	34	33
Data augmentation	516	65	66

**Table 2:** Hyper-parameter configuration for training detector.

	Hyper-Parameter Configuration		
Test	Max Epochs	Learning Rate	Optimizer
Test 1	20	0.001	adam
Test 2	20	0.001	sgdm
Test 3	20	0.001	rmsprop
Test 4	20	0.005	adam
Test 5	20	0.005	sgdm
Test 6	20	0.005	rmsprop
Test 7	20	0.0001	adam
Test 8	20	0.0001	sgdm
Test 9	20	0.0001	rmsprop
Test 10	20	0.001	adam
Test 11	40	0.001	sgdm
Test 12	40	0.001	rmsprop
Test 13	40	0.005	adam
Test 14	40	0.005	sgdm
Test 15	40	0.005	rmsprop
Test 16	40	0.0001	adam
Test 17	40	0.0001	sgdm
Test 18	40	0.0001	rmsprop

**Table 3:** ResNet-18 third molar angle detection results—mAP.

	Training			Validation			Testing		
Test	Faster R-CNN	Yolo V2	SSD	Faster R-CNN	Yolo V2	SSD	Faster R-CNN	Yolo V2	SSD
Test 1	2.3%	99%	83%	1.2%	85%	77%	2%	96%	75%
Test 2	25%	87%	60%	24%	61%	53%	39%	81%	51%
Test 3	1.3%	97%	84%	1%	84%	81%	1.3%	86%	77%
Test 4	3.2%	94%	84%	2%	71%	70%	3%	86%	69%
Test 5	5%	99%	86%	12%	85%	74%	14%	87%	72%
Test 6	8.4%	81%	3%	8.3%	63%	2.7%	7.1%	77%	2%
Test 7	6%	85%	88%	6%	72%	73%	5.2%	81%	71%
Test 8	3%	42%	1.4%	3%	38%	1.2%	2%	36%	1.3%
Test 9	4.6%	81%	88%	4.6%	70%	77%	3.6%	69%	74%
Test 10	9.3%	99%	88%	8.3%	90%	79%	8.3%	96%	77%
Test 11	16%	99%	89%	17%	82%	76%	12%	93%	75%
Test 12	6.2%	99%	87%	6%	84%	82%	4%	91%	81%
Test 13	6.3%	99%	88%	3%	92%	79%	8.9%	89%	78%
Test 14	11%	99%	88%	8%	88%	76%	19%	97%	77%
Test 15	2.2%	96%	87%	2%	84%	77%	1.5%	87%	78%
Test 16	18%	99%	89%	18%	85%	80%	28%	95%	79%
Test 17	14%	52%	6%	11%	50%	11%	21%	51%	8%
Test 18	8.2%	99%	88%	8.6%	79%	76%	8.6%	86%	76%

**Table 4:** ResNet-50 third molar angle detection results—mAP.

	Training			Validation			Testing		
Test	Faster R-CNN	Yolo V2	SSD	Faster R-CNN	Yolo V2	SSD	Faster R-CNN	Yolo V2	SSD
Test 1	6%	99%	88%	7%	85%	79%	19%	93%	77%
Test 2	18%	96%	91%	22%	87%	82%	24%	76%	82%
Test 3	3.3%	97%	86%	2.9%	81%	79%	3.1%	88%	78%
Test 4	8.2%	85%	84%	2%	72%	80%	7.2%	80%	72%
Test 5	17%	98%	1.3%	13%	89%	1.3%	17%	91%	1.2%
Test 6	1.2%	1.8%	1.5%	1.2%	1.5%	1.3%	1.1%	1.5%	1.4%
Test 7	16%	99%	87%	13%	89%	85%	14%	91%	85%
Test 8	8%	9%	65%	10%	8%	64%	13%	15%	63%
Test 9	7.6%	99%	88%	6.6%	91%	91%	7.1%	92%	88%
Test 10	14%	94%	88%	9%	82%	78%	21%	98%	78%
Test 11	35%	99%	89%	33%	90%	81%	34%	83%	79%
Test 12	3%	99%	86%	2.9%	88%	76%	2.9%	90%	79%
Test 13	8.2%	98%	87%	7.2%	78%	83%	8%	94%	82%
Test 14	30%	99%	2.1%	22%	84%	1.1%	18%	89%	1.2%
Test 15	4.2%	3%	2%	4%	2.6%	2.2%	4.2%	2.3%	2.5%
Test 16	12%	99%	89%	14%	90%	84%	17%	88%	84%
Test 17	23%	30%	82%	22%	20%	71%	20%	18%	70%
Test 18	8%	99%	88%	7.2%	89%	84%	6.9%	95%	83%

**Table 5:** ResNet-101 third molar angle detection results—mAP.

	Training			Validation			Testing		
Test	Faster R-CNN	Yolo V2	SSD	Faster R-CNN	Yolo V2	SSD	Faster R-CNN	Yolo V2	SSD
Test 1	2.8%	99%	88%	1.5%	90%	87%	2.1%	97%	87%
Test 2	20%	98%	86%	10%	81%	75%	24%	89%	72%
Test 3	1.8%	96%	87%	1.2%	79%	84%	1.1%	89%	84%
Test 4	7.2%	93%	64%	4.2%	84%	53%	3.1%	92%	52%
Test 5	28%	99%	3.2%	22%	86%	2.8%	35%	86%	3%
Test 6	1.6%	7%	3.3%	1.3%	12%	7.7%	1.1%	5%	2%
Test 7	20%	99%	88%	19%	92%	86%	27%	95%	84%
Test 8	12%	15%	66%	7%	11%	66%	25%	12%	62%
Test 9	3%	99%	89%	1.1%	93%	87%	2%	93%	82%
Test 10	9%	99%	85%	6%	87%	81%	1%	98%	81%
Test 11	21%	99%	88%	12%	92%	78%	14%	99%	77%
Test 12	5.5%	99%	88%	4%	83%	90%	3%	97%	89%
Test 13	3.3%	98%	88%	3%	80%	83%	2.8%	92%	77%
Test 14	23%	99%	5.2%	19%	85%	4.2%	26%	89%	4.1%
Test 15	6%	14%	2.9%	3%	9%	2%	6.5%	10%	2%
Test 16	18%	99%	88%	9%	91%	84%	31%	95%	81%
Test 17	16%	38%	82%	18%	20%	76%	33%	21%	74%
Test 18	2.7%	99%	88%	2.4%	92%	87%	2.1%	95%	83%

**Table 6:** Comparison of inference times between Faster R-CNN, YOLO V2, and SSD and their respective feature extraction methods.

Detection Model	ResNet-18 (s)	ResNet-50 (s)	ResNet-101 (s)
Faster R-CNN	0.223	0.188	0.360
YOLO V2	0.071	0.089	0.092
SSD	0.077	0.104	0.093

**Table 7:** Comparison results with state of the art works.

Paper	Objective	Algorithm	Feature Extractor	Data Base	mAp
Sathya et al. [47]	Third molar detection	AlexNet	AlexNet	106 Rx and 3159 tooth	97.5%
De Tobel et al. [18]	Third molar detection	DCNN	DCNN	400 Rx	51.0%
Lee et al. [15]	Caries detection	GoogleNet and Inception	GoogleNet and Inception	3000 Rx	89.0%
Tuzoff et al. [48]	Tooth identification	VGG-16	VGG-16	1352 Rx	99.5%
Jeon et al. [49]	Second molar canal detection	Xception	Xception	2040 Rx	93.6%
Cui et al. [49]	Tooth extraction detector	XGBoost	XGBoost	4135 Rx	87.9%
Takahashi et al. [50]	Dental Implant Detector	Yolo v3 (TensorFlow and Keras)	Yolo v3 (TensorFlow and Keras)	1282 Rx	85.0%
Lerner et al. [51]	Individual crown detection	Fully digital workflow	Fully digital workflow	106 Rx	91.3%
Vranckx et al. [52]	Third molar angle detection	CNN and ResNet-101	ResNet-101	838 Rx	98.1%
This Work	Third molar angle detection	Yolo v3, Faster R-CNN and SSD	ResNet-18, ResNet-50 and ResNet-101	644 Rx and 1604 molars	Yolo 98%, Faster 20 %, SSD 89%

## Data Availability

The raw data supporting the conclusions of this article will be made available by the authors on request.
